# Complex Genotype by Environment interactions and changing genetic architectures across thermal environments in the Australian field cricket, *Teleogryllus oceanicus*

**DOI:** 10.1186/1471-2148-11-222

**Published:** 2011-07-27

**Authors:** Magdalena Nystrand, Damian K Dowling, Leigh W Simmons

**Affiliations:** 1 School of Biological Sciences, Monash University, Clayton, 3800, Victoria, Australia; 2 Centre for Evolutionary Biology, School of Animal Biology (M092), University of Western Australia, Crawley, 6009, Western Australia, Australia

## Abstract

**Background:**

Biologists studying adaptation under sexual selection have spent considerable effort assessing the relative importance of two groups of models, which hinge on the idea that females gain indirect benefits via mate discrimination. These are the good genes and genetic compatibility models. Quantitative genetic studies have advanced our understanding of these models by enabling assessment of whether the genetic architectures underlying focal phenotypes are congruent with either model. In this context, good genes models require underlying additive genetic variance, while compatibility models require non-additive variance. Currently, we know very little about how the expression of genotypes comprised of distinct parental haplotypes, or how levels and types of genetic variance underlying key phenotypes, change across environments. Such knowledge is important, however, because genotype-environment interactions can have major implications on the potential for evolutionary responses to selection.

**Results:**

We used a *full diallel *breeding design to screen for complex genotype-environment interactions, and genetic architectures underlying key morphological traits, across two thermal environments (the lab standard 27°C, and the cooler 23°C) in the Australian field cricket, *Teleogryllus oceanicus*. In males, complex three-way interactions between sire and dam parental haplotypes and the rearing environment accounted for up to 23 per cent of the scaled phenotypic variance in the traits we measured (body mass, pronotum width and testes mass), and each trait harboured significant additive genetic variance in the standard temperature (27°C) only. In females, these three-way interactions were less important, with interactions between the paternal haplotype and rearing environment accounting for about ten per cent of the phenotypic variance (in body mass, pronotum width and ovary mass). Of the female traits measured, only ovary mass for crickets reared at the cooler temperature (23°C), exhibited significant levels of additive genetic variance.

**Conclusions:**

Our results show that the genetics underlying phenotypic expression can be complex, context-dependent and different in each of the sexes. We discuss the implications of these results, particularly in terms of the evolutionary processes that hinge on good and compatible genes models.

## Background

There has been a recent surge in interest among evolutionary biologists in elucidating the genetic determinants of phenotypic quality [1,2]. This has been driven in part by researchers working in the field of sexual selection, who have sought to address whether females might acquire indirect genetic benefits for their offspring by mating with males of particular genotypes. As such, much attention has focussed on two groups of models that centre on the genetic benefits that might be gained from exhibiting mate discrimination: *good genes *and *genetic compatibility *[3-12]. In short, good genes models are based on the premise that genetic variation, acting additively, underlies the phenotypes that contribute to fitness (for instance, those upon which females might base a mating preference) [5,7]. Thus, it is possible to rank males, according to their underlying genotypes, by their effect on the phenotype. In contrast, genetic compatibility models are based on the idea that no male genotype within a population is superior to others. Rather, mothers of certain genotypes will match better to sires of particular compatible genotypes, and thus, some male and female combinations will produce better phenotypes than others [8,9].

The contribution and relevance of good versus compatible genes in determining offspring genetic quality has been ardently debated for over a decade, but remains unclear [3-12]. This lack of clarity stems partly from historical confusion as to a robust theoretical and empirical framework with which to discern each process [5,13-15]. For instance, many tests of these models have relied on documenting phenotypic relationships between male sexual signals, mate preferences and subsequent offspring fitness [4]. However, it is clear that such phenotypic relationships will often provide misleading information as to the underlying genetic associations because of variation imposed by environmental factors [16,17].

In 2005, Neff and Pitcher outlined a quantitative genetic framework that enables a simultaneous assessment of the potential for good and compatible genes processes to operate within a given population. They pointed out [7], reinforced by others [5,6], that additive genetic variance for a given trait is consistent with the potential for good genes processes to operate, whereas non-additive genetic variance (encompassing dominance and epistatic variance) is consistent with the potential for genetic compatibility processes to operate. That is to say, that a particular allele that acts additively in effect on a given phenotype, acts independently of the other alleles with in the genome; whereas a particular allele that acts non-additively confers a given phenotypic value only in the presence of particular interacting alleles, either at the same locus (i.e. dominance) or at another locus (i.e. epistasis). Thus, if a particular phenotype possesses a strong underlying non-additive genetic component, then the expression of some key alleles that are transmitted from the father to the offspring will presumably be contingent on an associated set of alleles that are transmitted from the mother.

Several studies have now applied this quantitative genetic framework to assessing the potential for good and compatible genetic models simultaneously [18-28]. These studies have generally demonstrated that the genetic architecture of one or more key life history traits was consistent with the capacity to sustain either both of the models [19-25,27], or the compatibility model in particular [18,21,26]. Note however, that all of these studies have measured the focal trait in a common garden setting. Therefore, it remains unclear whether the underlying additive to non-additive genetic variances are robust to heterogeneity in environmental conditions of the type likely to be experienced by natural populations. That is to say, natural populations do not exist in common gardens, but have distributions that cover a range of environmental conditions that vary in space and time.

It is well known that the prevailing environment has a pervasive influence on the link between the genotype and the phenotype. This has been reinforced repeatedly across taxa and trait types (both morphological and life history), via studies showing that trait values associated with particular genes or genotypes frequently vary across environments [1,29-32]. Thus, for any given trait within any given species, we typically find variation in the slope of reaction norms that are associated with different genotypes [30]. The fact that genotype - environment interactions (GEIs) are a near ubiquitous phenomenon within populations also suggests that the amount, and perhaps type, of genetic variance underlying quantitative traits will vary across environments. Indeed, the available empirical evidence largely supports this suggestion, showing changes across environments in levels of additive and/or non-additive (dominance or epistasis) genetic variance for traits shaped by natural selection (e.g. body mass, wing size, development time and viability) [33-39]. For example, some studies have documented increases in genetic variance for traits expressed in stressful relative to control (standard rearing) environments [33,38,40], while others have showed no change [34,36], decreases [34,39], or variable patterns that change according to ontogenetic stage [41] or type of genetic variance [36]. Changes in the levels of genetic variance across environments have usually been attributed to novel gene expression in the new or stressful environment, or due to a historical absence of stabilizing selection on genetic polymorphisms when expressed at the novel environmental condition [38,42].

In this study we explore GEIs, and changes in genetic architecture across environments, in the expression of some classic fitness-related morphological traits in male and female Australian field crickets (*Teleogryllus oceanicus*). We take a quantitative genetic approach, applying an incomplete *full diallel *breeding design to eight inbred lines, generating 38 outbred F_1 _offspring genotypes consisting of various reciprocal combinations of parental haplotypes. F_1 _offspring of each genotype were then reared at two different temperatures until adulthood, when measures of body mass and size and gonad mass were obtained. This approach enables us to assess the reaction norms of each genotype across environments, examining whether the effect of the paternal haplotype, maternal haplotype, or the interaction between parental haplotypes, on each trait changes across environments. The design also enables us to partition the genetic variance underlying each trait into additive and non-additive contributions (separating them from the residual and environmental variance), and to examine the magnitude of any maternal and paternal effects across environments. We discuss the results in light of their potential to impact on good or compatible gene processes that might be operating within the studied population.

## Methods

### Study population and construction of inbred lines

The crickets used in the experiment were the immediate descendents of a sample (n > 100) of wild type adults collected from a banana plantation in Carnarvon, north-west Western Australia in 2006. The founder population was maintained in the laboratory at 27°C on a 12 h light: 12 h dark photoperiod in multiple replicate 10 L plastic containers, with *ad libitum *access to dry cat food and water. Ten females were randomly-sampled and used to create 10 separate isofemale lines. Each of these lines was then immediately subjected to a full-sibling inbreeding protocol, in which a single virgin daughter was mated to a full-sib brother each generation, over five sequential generations. The estimated inbreeding coefficient (F) after five generations of such inbreeding is 0.672. All inbred lines were reared across two separate 5 L containers for most of their development. At their penultimate moult into adulthood, they were sorted by sex (one container per sex).

### Diallel crosses

Inbreeding apparently affected productivity, with each line producing an average of only 14 pairs of adult offspring following five generations of inbreeding. This number was only sufficient to sustain an incomplete 8 × 8 full diallel reciprocal crossing scheme, performed in duplicate (the two weakest lines had to be discarded). Thus, for each of the remaining eight lines, a virgin adult dam was mated once to a virgin sire from one of the other seven lines, in all possible dam × sire line combinations. When possible (depending on available numbers of crickets), each line combination was replicated. We omitted inbred combinations from the crossing scheme - that is, we did not cross females from a given inbred line to males from the same line. After mating, each female was placed in a plastic container (16 × 12 × 5 cm) for two weeks (or until death), which contained food and a moist cotton pad for oviposition (replaced every 7 days).

The resultant nymphs of each replicate per line combination were distributed in equal densities across separate 5 L containers with *ad libitum *access to food and water. To mitigate potential density-dependent effects, we added two egg cartons to each container, and capped the maximum density of crickets at 30 per container. Nymphs were then exposed to one of two temperature treatments. Half of the nymphs per replicate line combination were placed at 27°C (standard rearing conditions for our laboratory culture) and the other half at 23°C. These temperature treatments fall well within the range of temperatures that *T. oceanicus *crickets experience within their native Australian distribution [43].

**Figure 1:**
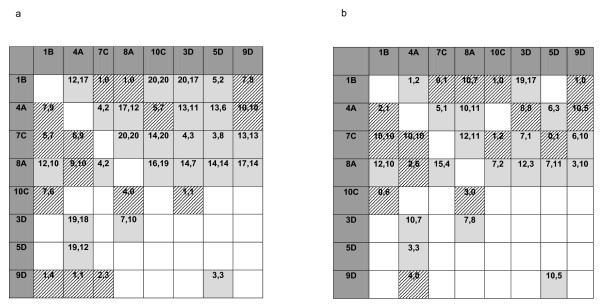
**The diallel crossing scheme represented at each thermal treatment: a) 27°C and b) 23°C**. Eight lines were crossed to each other in all combinations, barring the inbred combinations (female lines vertically, male lines horizontally). Cells shaded grey indicate those crosses that were duplicated, and hatched cells represent crosses without replication. The number of females versus males subsequently scored for fitness traits per cross are indicated within each cell. For example, in cross Dam 4A × Sire 1B at 27°C, 7 females and 9 males were sampled, with this cross not replicated (represented by the hatched cell).

We lost a number of cells from the diallel crossing scheme due to reproductive failure and mortality of the inbred parents involved in the crosses. This resulted in some crosses in the diallel not being represented at all, and others being represented by only one of the two replicates (Figure 1). Roff and Sokolovska [44] experienced similar problems when conducting diallel crosses in the sand cricket, *Gryllus firmus *(15 of 42 cells lost). Missing cells might affect the variance component estimates through a general loss of statistical power, or if the missing cells were distributed throughout the diallel in a non-random way. However, unless the missing cells consistently involved lines with intermediate breeding values (an unlikely scenario since it is likely that the weaker line combinations will have been the ones that failed to produce F_1 _offspring), then any bias in the genetic variance estimates will be in a downward direction, and our estimates can therefore be considered to be conservative [22,45].

### Measuring traits

As the F1 offspring reached their final moult, we sorted both focal sons and daughters into individual holding containers (7 × 7 × 5 cm), with *ad libitum *access to food and water. Focal individuals spent nine to 13 days in these containers, after which point they were frozen at - 20°C. Each focal daughter was provided with a male from the stock population with which to mate freely over six hours, and then frozen. We then measured a number of fitness-related morphological traits in up to 10 individuals of each sex per line combination and replicate (Figure 1). Using a Mettler Toledo AG245 micro-balance, we measured body mass, and ovary mass in females, and testes mass in males. We also measured pronotum width using digital callipers. Each of these traits is very likely to be linked to fitness in *T. oceanicus*, and in males these traits are potential targets of sexual selection. For instance, in the family Gryllidae crickets in general, male body size is frequently assumed to indicate male quality, given the links between body size and call frequency [46]. Furthermore, in the congeneric *T. commodus*, male body size determines fighting ability [47] and is under sexual selection [48]. While little is known about the genetic association between testes size and male fitness in *T. oceanicus *[49], studies employing experimental evolution in other insects have found that increasing the force of postcopulatory sexual selection in males (via an enforced polyandrous mating system relative to a monandrous system) can result in an evolutionary increase in testes size [50,51]. Finally, in female *T. oceanicus*, ovary mass is strongly associated with fecundity in females [49], but exhibits a negative genetic correlation with egg viability [52].

### Data analysis

We took a two-step approach to partitioning the phenotypic variance of each trait, allowing us to examine 1) complex GEIs in the phenotypic expression of each trait in the F_1 _offspring, and 2) the underlying genetic architecture of these traits when reared in the different thermal environments.

In all analyses, we tested male and female data separately, even for traits that are shared across sexes (i.e. body mass and pronotum width). This is because these traits are sexually dimorphic (body mass F_1,1042 _= 11.92 p = 0.0006; pronotum width F_1,1043 _= 358.91 p = < 0.0001, Table 1), and the inclusion of both sexes into the one model introduced complex higher order (4-way) interactions, which resulted in model overfitting. Moreover, previous studies of body size [53], life span [54,55] and thermal tolerance traits [56] in *D. melanogaster *indicate that the genetic architecture of each of these traits differs across the sexes. These findings reinforce our decision to analyze pronotum width and body mass separately by sex in this study.

**Table 1:** Trait means (± SE) and sample sizes for females and males, in each temperature treatment (H = 27°C, L = 23°C).

Trait	Sex	Mean (SE) H	n	Mean (SE) L	n
Pronotum width	♀	6.061 (0.013)	352	5.952 (0.022)	214
Pronotum width	♂	6.376 (0.016)	333	6.632 (0.023)	171
Body mass	♀	0.727 (0.005)	352	0.642 (0.007)	214
Body mass	♂	0.683 (0.004)	331	0.643 (0.007)	171
Ovary mass	♀	0.137 (0.002)	350	0.092 (0.002)	213
Testes mass	♂	0.032 (0.0003)	332	0.031 (0.0004)	171

To examine GEIs on phenotypic expression, we estimated the variance component s associated with the parental Sire and Dam lines, and the Dam × Sire line interaction in both thermal treatments (23°C or 27°C), for each focal trait. This was done using Restricted Maximum Likelihood Analysis (REML) in the MIXED procedure in SAS (v. 9.1, SAS Institute Inc., Cary, USA). Sire and Dam effects were treated as random effects in the model, with individual line combination replicates nested within Sire × Dam combinations. The thermal treatment was treated as a fixed effect, and all interactions as random effects.

To discern the underlying genetic architecture of each trait at each thermal treatment, we applied the *Bio model *described by Cockerham and Weir [57] and Lynch and Walsh [58] to the data. This enabled us to break down the variance components into additive and non-additive genetic contributions, and separate out maternal and paternal contributions. The model equation for this procedure was:

where *Y_ijkl _*is the trait of the l'th offspring from the *k*'th replicate of the cross between sire *i *and dam *j*, and μ = the trait mean of the population. *N_i _*and *N_j _*are the additive effects of the nuclear genes contributed by *i *and *j*, independent of sex; *T_ij _*is the interaction of the haploid nuclear contributions; *Mj *is the maternal genetic and environmental effects of line *j *when used as dams; *Pi *is the paternal genetic and environmental effects of line *i *when used as sires; *Kij *is the interaction between maternal and paternal effects; *Rk(ij) *is the effect of *k*'th replicate cross within the dam line × sire line combination and *Wl(k(ij)) *is the unique effect of individual *l *within a replicate cross [22,23,59]. These genetic variance components were again estimated by Restricted Maximum Likelihood Analysis (REML), using the MIXED procedure in SAS v.9.1.3 (SAS Institute 2004), and the TYPE = LIN command to model the covariance between families as linear functions of the variances. The analysis was performed on the matrix of between-line crosses (excluding the within-line crosses). We tested the one-sided hypotheses that parameter estimates are larger than 0 with likelihood ratio tests, by comparing models where a given parameter was set to 0 with a model where all parameters were allowed to assume non-negative values [see [59] for details of this model and associated example script]. In addition to the script outlined in Fry (2004), we included a statement that separates the variance among individuals within replicate crosses from the variance among replicate crosses. We then estimated the causal components of the observational variance components using the approach outlined in Fry (2004) and Bilde et al. (2008).

1) σ^2^*_n_*: nuclear additive variance = 1/2 FV_A _+ 1/4 F^2^V_AA_, where V_A _is the additive genetic variance, and V_AA _the additive-by-additive epistatic variance, and higher order epistasis is ignored for simplicity [60]. Thus, to enable us to estimate the causal component of additive variation, for simplicity we will assume that V_AA _is small, and V_A _can then be estimated as 2σ^2^*_n _*/F. *Consistent with good genes models*.

2) σ^2^*_t_*: nuclear interaction variance = 1/2 F^2^V_AA _+ F^2^V_D _+ F^3^V_AD _+ F^4^V_DD_, where V_AD _and V_DD _are the additive-by-dominance and dominance- by-dominance epistatic variances. Thus, the term includes epistatic and dominance variation. Again, if we make the assumption that the epistatic terms are small, then we can estimate V_D _as σ^2^*_t_*/F^2^. *Consistent with compatible genes models*.

3) σ^2^*_m_*: maternal effect variance V_M_, including both maternal genotype and environment effects, and possible interactions between maternal nuclear and maternal extra-nuclear effects.

4) σ^2^*_p_*: paternal effect variance V_P_, including both paternal genotype and environment effects, and possible interactions between paternal nuclear and paternal extra-nuclear effects. Significant paternal effect variance might in theory be consistent *with good genes models under the assumption that the paternal effects are genetic in origin*.

5) σ^2^*_k_*: non-reciprocal interaction variance, V_K_, of paternal and maternal effects, and of nuclear and extra-nuclear effects. *Consistent with compatible genes models under the assumption that the non-reciprocal interaction variance is genetic in origin*.

6) σ^2^*_rep _*: variance among replicate crosses within line combinations.

7) σ^2^*_w_*: variance among individuals within replicate crosses.

Coefficients of genetic variation (CV) for each variance component estimate were calculated by scaling the causal variance components by the trait mean; e.g. CV_A _= 100(√V_A_)/trait mean [61].

All traits exhibited normal distributions of raw data and residuals, with few if any outlying datapoints.

## Results

### Genotype × Environment Interactions

**Figure 2:**
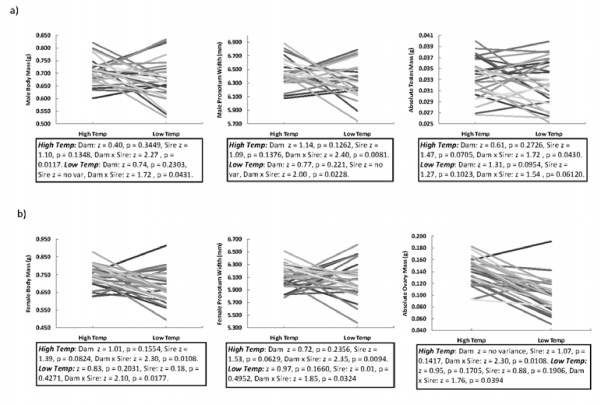
**Interaction plots, showing the mean trait values and reaction norms for each Dam × Sire line combination across each thermal environment (27° vs 23°C) for each trait type in a) males and b) females**. Thus, each line represents a distinct Dam × Sire genotype. Below each plot, we present the statistics for each environment separately (i.e. Dam and Sire effects within each thermal treatment).

In sons, all traits measured (pronotum width, body mass & testis mass) were affected by a Sire × Dam × Treatment effect, explaining between 16 and 23 per cent of the phenotypic variance in those traits (Table 2, Figure 2). That is, phenotypic expression in the male F_1 _offspring was typically determined by complex interactions involving each parental haplotype and the environment in which these haplotypic combinations were expressed. A further analysis of Sire and Dam effects within each of the two thermal environments shows that Sire × Dam interactions abound in each environment, for most of the examined traits (see test statistics presented within Figure 2), involving substantial crossing of reaction norms (Figure 2). In contrast, two-way GEIs (i.e. Sire × thermal treatment, Dam × thermal treatment) did not significantly contribute to phenotypic expression of the son morphologies, nor were there any substantial main effects of Sire or Dam line that were general across both environments. Thus, the expression of each male trait was affected by an interaction between the Sire and the Dam lines used, but this effect was itself contingent in part on whether the traits were assayed at 27°C or at 23°C.

**Table 2:** Effects of Sire and Dam lines, thermal Treatment, Replicate (Repl), and their interactions, on the expression of the offspring morphological traits.

		*Estimate*	*SE*	*F*	*p-value*	*Percent*
	
	***Treatment***	*F_1,11 _= 1.70*	*P = 0.2191*			
	
	Dam	0	.	.	.	0
	Sire	0.004325	0.005812	0.74	0.2284	4.82760
	Dam * sire	0	.	.	.	0
	Dam * Treatment	0.009832	0.007858	1.25	0.1054	10.97456
Pronotum Width (male)	Sire * Treatment	0	.	.	.	0
	Dam * Sire * Treatment	0.02045	0.008901	2.30	0.0108	22.82646
	Repl (dam * sire)	0.004050	0.004928	0.82	0.2056	4.52064
	Treatment * Repl (dam*sir)	0.004772	0.006953	0.69	0.2462	5.32655
	Residual	0.04616	0.003236	14.27	<.0001	51.52418
	***Treatment***	***F_1,7.33 _= 2.95***	***P = 0.1277***			
	
	Dam	0	.	.	.	0
	Sire	0.000522	0.000550	0.95	0.1717	6.90020
	Dam * sire	0	0	.	.	0
**Body Mass (male)**	Dam * Treatment	0.000459	0.000621	0.74	0.2301	6.06742
	Sire * Treatment	3.39E-53	.	.	.	0
	Dam * Sire * Treatment	0.001596	0.000783	2.04	0.0208	21.09716
	Repl (dam * sire)	0	.	.	.	0
	Treatment * Repl (dam*sire)	0.000798	0.000542	1.47	0.0703	10.54858
	Residual	0.004190	0.000296	14.17	<.0001	55.38665

	***Treatment***	***F_1,2.78 _= 2.78***	***P = 0.2222***			
	
	Dam	4.158E-6	3.879E-6	1.07	0.1419	14.28621
	Sire	6.728E-6	4.599E-6	1.46	0.0717	23.11630
	Dam * sire	0	.	.	.	0.00000
**Testis Mass**	Dam * Treatment	1.375E-6	2.417E-6	0.57	0.2847	4.72427
	Sire * Treatment	4.35E-7	1.685E-6	0.57	0.2847	1.49459
	Dam * Sire * Treatment	4.539E-6	2.261E-6	2.01	0.0223	15.59526
	Repl (dam * sire)	3.38E-7	1.394E-6	0.24	0.4042	1.16131
	Treatment * Repl (dam*sire)	1.532E-6	1.861E-6	0.82	0.2051	5.26370
	Residual	0.000010	0	.	.	34.35836

	***Treatment***	***F_1,12.6 _= 0.15***	***P = 0.7046***			
	
	Dam	.	.	.	.	0
	Sire	0	.	.	.	0
	Dam * sire	0	.	.	.	8.19016
**Pronotum width (female)**	Dam * Treatment	0.007346	0.006882	1.07	0.1429	13.56851
	Sir * Treatment	0.01217	0.008006	1.52	0.0643	7.30157
	Dam * Sire * Treatment	0.006549	0.009757	0.67	0.2511	4.25674
	Repl (dam * sire)	0.003818	0.006471	0.59	0.2776	23.44665
	Treatment * Repl (dam*sire)	0.02103	0.01195	1.76	0.0392	43.23637
	Residual	0.03878	0.002572	15.08	<.0001	0

	***Treatment***	***F_1,12 _= 6.16***	***P = 0.0289***			
	
	Dam	0	.	0.46	0.3223	0
	Sire	0	.	.	.	0
	Dam * sire	0.000068	0.001001	0.07	0.4728	0.69772
**Body Mass (female)**	Dam * Treatment	0.000449	0.000526	0.85	0.1967	4.60702
	Sire * Treatment	0.001171	0.000812	1.44	0.0746	12.01519
	Dam * Sire* Treatment	0.000735	0.001667	0.44	0.3296	7.54156
	Repl (dam * sire)	0.000549	0.000956	0.57	0.2827	5.63308
	Treatment * Repl (dam*sire)	0.002090	0.001397	1.50	0.0673	21.44470
	Residual	0.004684	0.000313	14.96	<.0001	48.06074

	***Treatment***	***F_1,11.8 _= 24.65***	***P = 0.0003***			
	
	Dam	0	.	.	.	0
	Sire	0	.	.	.	0
	Dam * sire	0.000071	0.000113	0.63	0.2369	4.80704
**Ovary Mass**	Dam * Treatment	0.000042	0.000046	0.93	0.1775	2.84360
	Sire * Treatment	0.000159	0.000095	1.68	0.0463	10.76506
	Dam * Sire * Treatment	0	.	.	.	0
	Repl (dam * sire)	0.000215	0.000134	1.61	0.0535	14.55653
	Treatment * Repl (dam*sire)	0.000052	0.000076	0.68	0.2470	3.52065
	Residual	0.000938	0.000062	15.10	<.0001	63.50711

Sire and Dam lines were treated as Random effects in the model and Thermal Treatment as a fixed effect. Fixed effect parameter estimates are displayed above the hatched lined for each trait (in italics). The percentage of total phenotypic variance explained by each random effects term and interaction is denoted in the right hand column of the table.

In daughters, three way interactions between parental haplotypes and the rearing environment were not significant. Rather, the female traits measured (pronotum width, body mass and ovary mass), were influenced by Sire × Treatment interactions that explained seven to 12 per cent of the phenotypic variation in those traits (Table 2).

### Genetic architectures

**Figure 3:**
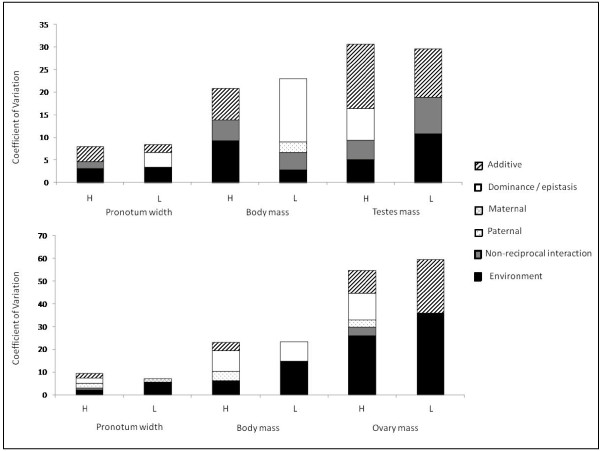
**Coefficients of variation (CVs) for variance components underlying each trait across each thermal environment (H denotes 27°C and L denotes 23°C)**. Male traits are represented on the top graph, and female traits on the lower graph.

The *Biomodels *revealed significant additive genetic variance components (σ^2^*_n_*) underlying all of the male phenotypes (Table 3) when reared at the laboratory standard (27°C) thermal treatment. Coefficients of additive genetic variance ranged from three to 14 (Table 4). In contrast, we detected less additive genetic variance for these traits when crickets were measured at the cooler thermal treatment (Table 3, 4, Figure 3). Only male testis mass exhibited a relatively stable genetic architecture across environments. Additionally, male body mass and pronotum width exhibited significant non-reciprocal interaction variance components (σ^2^*_k_*) in the standard, but not cooler thermal treatment (Table 3 and 4). We found no significant underlying dominance or epistatic genetic variance in any of these traits.

**Table 3:** Estimates of raw observational variance estimates for male traits, including standard errors (SE).

		*Male pronotum width*			*Male Body Mass*			*Testes Mass*		
	***Variance component***	***Estimate***	***SE***	***p***	***Estimate***	***SE***	***p***	***Estimate***	***SE***	***p***
	
	σ^2^*n*	**0.01422**	**0.00932**	**0.00050**	**0.000776**	**0.00058**	**0.01352**	**7.01E-06**	**4.50E-06**	**0.00552**
	
	σ^2^*t*	0	.	1	0	.	1	2.32E-06	2.43E-06	0.75183
	
*High Temperature*	σ^2^*m*	0	.	1	0	.	1	0	.	1
	
	σ^2^*p*	0	.	1	0	.	1	0	.	1
	
	σ^2^*k*	**0.01111**	**0.00625**	**0.02535**	**0.00097**	**0.00054**	**0.04042**	1.88E-06	1.75E-06	0.14730
	
	σ^2^_rep_	0.00663	0.00434	0.01603	0.00052	0.00039	0.05778	1.34E-06	0	0.03016
	
	σ^2^*w_1_*	0.04415	0.00371	-	0.00424	0.00036	-	0.000011	0	-

	σ^2^*n*	0.00042	0.008626	0.58388	1.76E-20	.	1	3.70E-06	3.22E-6	0.08327
	
	σ^2^*t*	0.01869	0.02544	0.43858	0.00371	0.002220	0.22065	0	.	1
	
*Low Temperature*	σ^2^*m*	0	.	1	0	.	1	0	.	1
	
	σ^2^*p*	0	.	1	0.00021	0.000694	0.75183	0	.	1
	
	σ^2^*k*	0	.	1	0.00062	0.001652	0.58388	6.05E-06	5.96E-6	0.31731
	
	σ^2^_rep_	0.02288	0.02108	0.15730	8.41E-20	.	1	5.60E-06	4.89E-6	0.10035
	
	σ^2^*w_1_*	0.04879	0.006302	-	0.00484	0.000584	-	9.17E-06	1.14E-6	-

Displayed is also the contribution that each variance component had on the model (bold writing indicates variance components that significantly contributed to the model).

**Table 4:** Estimates of raw Causal Variance estimates and the Coefficient of Genetic Variation (*CV*) for male traits.

		*Male pronotum width*			*Male Body Mass*			*Testes Mass*		
	
	*Variance component*	*Estimate*	*%*	*CV*	*Estimate*	*%*	*CV*	*Estimate*	*%*	*CV*
	*V*_A_	**0.04233**	**46.86177**	**3.22700**	**0.00231**	**31.74757**	**7.04054**	**2.09E-05**	**68.26233**	**14.23191**
	
	*V*_D_	0	0	0	0	0	0	5.13E-06	16.80276	7.06095
	
*High Temperature*	*V*_M_	0	0	0	0	0	0	0	0	0
	
	*V*_P_	0	0	0	0	0	0	2.60E-22	0	0
	
	*V*_K_	**0.01111**	**12.29962**	**1.65322**	**0.00097**	**13.31776**	**4.56002**	1.88E-06	6.15772	4.27448
	
	*V*_E_	0.03689	40.83861	3.01245	0.00400	54.93467	9.26135	2.68E-06	8.77719	5.10329

	*V*_A_	0.01239	12.55166	1.78559	5.24E-20	5.59E-16	0	0.00001	39.0019	10.80332
	
	*V*_D_	0.04140	41.95603	3.26459	0.00822	87.59454	14.09379	0	0	0
	
*Low Temperature*	*V*_M_	0	0	0	0	0	0	0	0	0
	
	*V*_P_	0	0	0	0.00021	2.260128	2.26389	0	0	0
	
	*V*_K_	0	0	0	0.00062	6.641791	3.88090	0.00001	21.44351	8.01055
	
	*V*_E_	0.04489	45.49231	3.39938	0.00033	3.503542	2.81866	0.00001	39.55459	10.8796

Bold writing indicates variance components that significantly contributed to the model.

In contrast to males, only one of the female traits (ovary mass) displayed a significant additive genetic variance component (CV = 23.5: Table 5 and 6, Figure 3), but this genetic variance was only detected in the cooler thermal treatment. In addition, female body mass exhibited a significant nuclear interaction variance σ^2^*_t_*) in the high temperature treatment. If we assume no epistasis, then we can estimate the coefficient of dominance variance for this trait at 9.2 (Table 6, Figure 3). Furthermore, female pronotum width exhibited paternal variance (σ^2^*_p_*), with a related CV of 1.9 (Table 5 and 6, Figure 3), but again, only in the warmer temperature treatment.

**Table 5:** Estimates of raw observational variance estimates for female traits, including standard errors (SE).

		*Female pronotum width*			*Female Body Mass*			*Ovary Mass*		
	***Variance component***	***Estimate***	***SE***	***p***	***Estimate***	***SE***	***p***	***Estimate***	***SE***	***P***
	
	σ^2^*n*	0.00418	0.00472	0.27332	0.00026	0.00053	0.58388	0.00006	0.00010	0.43858
	
	σ^2^*t*	0.00958	0.00718	0.12937	**0.00204**	**0.00104**	**0.00053**	0.00012	0.00014	0.3428
	
*High Temperature*	σ^2^*m*	1.48 E-19	.	1	0	.	1	0	.	1
	
	σ^2^*p*	**0.01322**	**0.00974**	**0.01603**	0.00087	0.00080	0.43858	0.00002	0.00008	0.74183
	
	σ^2^*k*	0.00281	0.00408	0.40278	0	.	1	0.00003	0.00015	1
	
	σ^2^_rep_	0.00304	0.00255	0.10686	0.00072	0.00041	0.00815	0.00019	0.00014	0.05125
	
	σ^2^*w_1_*	0.03114	0.00257	-	0.00399	0.00033	-	0.00129	0.00011	-

	σ^2^*n*	0	.	1	0	.	1	**0.00016**	**0.00013**	**0.03390**
	
	σ^2^*t*	0	.	1	0.00132	0.00203	0.52709	0	.	1
	
*Low Temperature*	σ^2^*m*	7.14E-03	0.01318	0.43858	0	.	1	0	.	1
	
	σ^2^*p*	0	.	1	0	.	1	0	.	1
	
	σ^2^*k*	0	.	1	0	.	1	0	.	1
	
	σ^2^_rep_	0.05857	0.01939	<0.001	0.00500	0.00242	<0.001	0.00073	0.00026	<0.001
	
	σ^2^*w_1_*	0.0511	0.00555	-	0.00583	0.00065	-	0.00052	0.00006	-

Displayed is also the contribution that each variance component had on the model (bold writing indicates variance components that significantly contributed to the model).

**Table 6:** Estimates of raw Causal Variance estimates and the Coefficient of Genetic Variation (*CV*) for female traits.

		*Female pronotum width*			*Female Body Mass*				*Ovary Mass*	
	
	*Variance component*	*Estimate*	*%*	*CV*	*Estimate*	*%*	*CV*	*Estimate*	*%*	*CV*
	*V*_A_	0.012434	18.24672	1.839665	0.00078	9.589413	3.838913	0.00191	10.75729	10.05514
	
	*V*_D_	0.021227	31.14999	2.403673	**0.004508**	**55.42895**	**9.22955**	0.000266	15.01017	11.87762
	
*High Temperature*	*V*_M_	1.48E-19	2.17E-16	6.35E-09	0	0	0	0	0	0
	
	*V*_P_	**0.01322**	**19.40038**	**1.896931**	0.000867	10.66027	4.047589	0.00002	1.07284	3.17544
	
	*V*_K_	0.002811	4.125149	0.874714	0	0	0	0.00003	1.46810	3.71462
	
	*V*_E_	0.018452	27.07776	2.241056	0.001978	24.32136	6.113728	0.00127	71.6916	25.95796

	*V*_A_	0	0	0	0	0	0	**0.000464**	**29.74837**	**23.50375**
	
	*V*_D_	0	0	0	0.002931	24.12163	8.427281	0	0	0
	
*Low Temperature*	*V*_M_	7.14E-03	6.114902	1.419851	0	0	0	0	0	0
	
	*V*_P_	0	0	0	0	0	0	0	0	0
	
	*V*_K_	0	0	0	0	0	0	0	0	0
	
	*V*_E_	0.10967	93.8851	5.563476	0.009219	75.87837	14.94663	0.00110	70.25163	36.11883

Bold writing indicates variance components that significantly contributed to the model.

## Discussion

Complex GEIs (haplotype × haplotype × environment) existed for all three of the male traits that we measured. In contrast, female phenotypes were in part shaped by interactions involving only the sire haplotype and environment. Furthermore, the relative contributions of additive and non-additive genetic variance underlying these same traits commonly changed across environments. We suggest that these results are likely to have implications on our understanding of several evolutionary concepts.

### Genotype × Environment Interactions

Firstly, our results indicate that trait expression in male and female offspring is influenced by different levels of genetic complexity. In males, all traits were generally shaped by the specific combination of haplotypes that the offspring inherit from their parents in interaction with the environment; whereas in females, all traits were influenced by the sire effect only in interaction with the environment. Notably, across both sexes, the genetic effects were completely contingent on the environment in which the offspring were reared in. That is, in no case did we find Sire × Dam effects on F_1_trait expression that were general across environments. Likewise, in no case did we find additive main effects of the maternal or paternal haplotype on F_1 _trait expression that were general across environments.

The general lack of main effects of the parental haplotype on offspring phenotypes, and the prevalence of paternal haplotype × environment interactions for female traits, and the more complex paternal haplotype × maternal haplotype × environment interactions for male traits, adds a previously underappreciated level of complexity to our understanding of evolutionary dynamics within populations. The fact that no particular paternal haplotype resulted in greater phenotypic expression across all maternal haplotypes and environments is consistent with a resolution to the Lek Paradox (i.e. how genetic variation is maintained under directional selection) based on GEIs [62-68], as well as to the general question of whether non-additive gene interactions can contribute to maintaining genetic diversity within populations [69,70]. A key assumption of the Lek Paradox is that traits subject to strong selection will initially harbour additive genetic variance that is eroded over time - unless there is a process that can counter this erosion. In line with this assumption, and acknowledging that GEIs might provide a resolution to the Paradox, we found that all male traits (likely to be under strong selection) harboured additive genetic variance (at least when measured at the standard thermal treatment), and that they were also all involved in G × E interactions. However, on the other hand, the existence of complex GEIs might well reduce the ability of females to accurately discriminate between males on the basis of acquiring good genes for their offspring, [1,68,71], particularly if the rank order in performance of a given set of male genotypes, for a given trait, changes dramatically across environments. This is because the consequences to a female of mating with a male of a particular genotype (in terms of the expression of the offspring traits measured here) will hinge on the genotype of the female involved as well as the thermal environment under which their offspring will develop. This level of complexity will presumably decrease, although not necessarily eliminate, the potential benefits to females of mate discrimination for good genes benefits, especially if there are costs involved in such choice.

Haplotype × haplotype × environment interactions are arguably more consistent with genetic compatibility models of quality, and it is worth asking whether such interactions might promote the evolution of mechanisms that allow females to discriminate between males on the basis of their genetic compatibility. Genetic compatibility models have risen to prominence in recent times [5,7-10], with many quantitative genetic examples to suggest that Sire × Dam interactions are prevalent [18,20-26], at least for juvenile life history traits (such as juvenile survival and development). One point worth noting is that there has previously been very little, if any, empirical attention devoted to examining whether Sire × Dam interactions (hence evidence for genetic compatibility) affect the expression of adult life history traits (reproductive parameters, morphologies and survival). In the context of the complex GEIs that we have identified in this study, if genetic compatibility models based on mate choice were to be relevant to this population of crickets in terms of offspring body and gonad size, then females would not only have to assess the quality of a focal male in relation to her own genotype, but also in relation to the environment in which the female will produce her offspring. This might therefore require the females to predict the environment in which the offspring will develop. The likelihood of such a scenario has to be assessed against how well our experimental set up reflects the natural environment of the crickets.

The thermal treatments that we used fall within the thermal range of the native distribution of *T. oceanicus *[43], as well as within the range of natural seasonal variation at the field collection site of these crickets in Canarvon, Western Australia. Given that *T. oceanicus *breeds year round [72,73], it is realistic to expect that adults and developing nymphs in the wild at Carnarvon might experience temperatures within the range of our thermal treatments. Thus, our experiment shows that complex haplotype × environment interactions can indeed affect the expression of offspring phenotypes when thermal environments differ consistently by a few degrees, and such temperature differences may well be represented in the wild. That said, it is highly likely that crickets in the field are able to better offset variation in thermal environments through microhabitat selection than they are when in the lab (the crickets live in cracks in the ground or deep in the leaf litter where the microclimate remains warm). But note however, that we are unable to use this result to predict whether smaller thermal differences, or fluctuating temperatures, will affect the expression of parental haplotypes to the same extent. Further research is thus required to assess how the reaction norms tied to particular parental haplotypes respond to more subtle changes in the prevailing environment.

Although the traits that we assessed in this study were all morphological, the available evidence suggests that each is under selection and closely tied to fitness in males and females (see Methods). In this regard, these traits therefore provided a good opportunity to assess the relevance of genetic models, such as the *good *and *compatibility *genetic models to phenotypic expression, particularly given the paucity of information that currently exists as to the relevance of genetic compatibility models on adult trait expression (almost all previous studies have examined compatibility effects on juvenile fitness components). Models of good genes and genetic compatibility are almost always posed in the context of sexual selection, typically addressing the question of whether females might acquire good or compatible genes through mate choice. Therefore, it is worth asking whether the set of morphological traits that we measured might be used in mate choice or male-male competition for matings. Body and pronotum size are clearly traits whose expression might be readily assessed by the opposite sex, and as previously mentioned, studies in the closely related species *T. commodus *show that male body size is indeed under sexual selection [47,48]. On the other hand, there is no evidence for an effect of male body size on fighting ability in *T. Oceanicus *[74]. Body and pronotum size in females, however, is under natural selection (large females produce larger ovaries, r = 0.37 [49], thus more eggs [49,52]. On the other hand, gonadal traits in crickets lie embedded within the body cavity where they cannot be directly assessed during mate choice. However, testes size is clearly under sexual selection in several species [50,51], and its expression is phenotypically correlated to body size in *T. oceanicus *(r = 0.37, n = 504), thereby providing females with a mechanism by which to choose males on the basis of large ejaculate investment. Moreover, molecules produced by the testes clearly have important downstream effects on female reproductive biology in *Teleogryllus*. In *T. Commodus*, the testes generate an enzyme complex, Prostaglandin synthetase, that contributes to the production of the chemical messenger Prostaglandin, which is transferred to the females in the seminal fluid [75]. These prostaglandins exert strong physiological effects on females, for example by stimulating oogenesis and elevating oviposition [76,77], as well as by accumulating within the female's neural tissue (specifically in the head region [78], and possibly also suppressing the phonotaxis response to sexually signalling males [77]). Hence, we conclude that all of the morphological traits used in this study were relevant candidates for genetic quality models based on mate choice.

### Genetic architectures

When it came to assessing the genetic architectures of the traits at hand, we generally found additive genetic variance for each male trait in the lab standard thermal environment (27°C), but not in the cooler environment (23°C). However, in females, we generally detected no such additive variance, with the exception of ovary mass at 23°C. This result is generally encouraging for good genes models based on mate choice, because it suggests that male candidate traits that are under sexual selection also exhibited an underlying genetic architecture consisting of standing additive variance. The disclaimer is that this genetic additively was only detected at 27°C, meaning that females might only draw benefits of discriminative mate choice if they can control or predict the environments in which their offspring are raised.

Previously, Simmons (2003) used a paternal half-sibling analysis to measure the coefficients of additive genetic variance in male testes and pronotum width, and female ovary mass and female pronotum width in *T. oceanicus*. While Simmons's results for pronotum width were consistent with our results in males and females [49], the measured CV_As _for testes and ovary mass were different in magnitude to those measured in the current study. Specifically, at similar temperatures to that of the high temperature treatment in this study, Simmons found lower levels of additive variance for testis mass (CV_A _= 7.18 compared to 14.23). In contrast, he did find higher additive variance for ovary mass than we did in our study (32.05 vs. 10.06). These discrepancies are not surprising, and can be reconciled by considering that the two studies were separated in time by about a decade, and each study exhibited differences in their respective technical designs (e.g. in the breeding designs employed, the age and mating history of the focal individuals, and in sample size of captured genotypes from the wild type population).

As mentioned above, almost all of the detectable significant genetic variance for the morphological traits was found in the 27°C treatment, and we detected only CV_A _for ovary mass at 23°C. Although 27°C represented the standard rearing condition for crickets in our study, the crickets had only recently been sourced from the field population where they breed year round [72], and had not had any chance to adapt to the new rearing conditions (fewer than six generations in the lab under a heavy inbreeding protocol that meant that the genotypes were thus shaped under drift, not selection). As outlined within the Introduction, several studies prior to ours have detected differences in genetic architectures across environments [33-39], usually attributing one of the environments as the stressful or novel environment, and the other as the benign or standard environment. Given *T. oceanicus *breeds all year round in the field, it is clear that neither of the treatments is novel to the crickets, although it is likely that the cooler temperature was more stressful given that peak cricket abundances in the field occur during the warmer seasons [73]. Finally, we point out that the sample sizes differed across treatments, with slightly fewer cells and replicates represented in the diallel cross at 23°C (Figure 1). This might have resulted in a general reduction of statistical power in the cooler treatment.

### Reconciling the quantitative genetic and GEI analyses

The presence of haplotype × haplotype × environment interactions, and haplotype × haplotype interactions within each environment (see test statistics in Figure 2) suggested that we should have also found abundant levels of non-additive genetic variance underlying the morphological traits in each environment. This was not the case. Our non-additive genetic variance components were represented by σ^2^t, an estimate of the reciprocal nuclear interaction variance (dominance or epistatic genetic variance), and σ^2^k, an estimate of complex non-reciprocal non-additive variance (including possible interactions of paternal and maternal effects, and of nuclear and extra-nuclear effects; see methods). Breaking down the non-additive variance components into these two estimates provided us with a more detailed picture of the genetic architecture of the traits at hand. The consequence of doing this, however, is that it decreases the power of finding significant non-additive variance components (relative to other studies based on North Carolina II breeding designs that have used the Sire term to directly estimate additive genetic variance, and the Dam × Sire interaction to directly estimate the amount of non-additive genetic variance [24,26-28]. This explains the discord between our GEI and the *Biomodel *results. In contrast, the *Biomodel *actually has greater power to detect additive genetic variance than similar models that simply use the Sire effect as an indicator of additive genetic variance (as in the GEI analyses), because it uses information from both sires and dams to calculate the additive variance [59]. Accordingly, while we did not find additive sire effects at 27°C for any of the male traits measured (as indicated by the Sire × Treatment terms), we did detect additive genetic variance for all male traits at 27°C using the *Biomodels*. This indicates that additive variance is indeed present, but is swamped by more complex Sire × Dam × Treatment interactions in the GEI analyses.

### Relevance to the natural world

Our study offers valuable insights into the likely complexity of gene by environment interactions in natural populations, and the evolutionary implications of these interactions. To generate these insights, we originally captured eight random parental genotypes from a wild population of *T. oceanicus*, and used these to generate 36 focal genotypes. Although these 36 genotypes represent a random sample of the total genetic variation segregating within the *T. oceanicus *population in question, it is important to realize that these genotypes nonetheless constitute only a tiny fraction of the total pool of possible genotypes within the population. This has implications. First, it is entirely possible that the parental genotypes that acted additively in our set of diallel lines, may not act additively in the wild population when matched against the complete pool of possible genotypes. Similarly, genotypes acting non-additively in our sample of genotypes, might exhibit additivity among the total pool of genotypes [6]. Although this point is pertinent to most quantitative genetic studies, it is particularly relevant to diallel designs which start with a small set of parental genotypes. Second, in diallel breeding designs, the parents used are inbred (although the F1 offspring genotypes that are subsequently tested are completely outbred). This might mean that presence and magnitude of parental effects are not representative of the magnitude of effects that occur in wild populations, in which the parents are likely to be outbred. Thus, while the results we present are accurate for the specific combinations of haplotypes used here, we must be cautious when scaling these results up to the genetic effects that occur within the wider natural population, let alone the species. Having said that, our motivation was not to calculate the absolute value of each genetic variance component to each trait in this species, but rather to address the potential magnitude by which genetic variances and gene expression might vary across environments.

## Conclusions

In sum, we have demonstrated that complex GEIs make a significant contribution to offspring phenotype expression in *T. oceanicus*, and this result suggests that genetic compatibility models might be relevant to overall genetic quality. However, our quantitative genetic *Biomodels *also show that there is some additive genetic variance underlying key male traits that are likely shaped by sexual selection. Thus, the combined results suggest that there is at least the potential, genetically, for females to utilize a good and/or a compatible genes process when choosing mates to procure genetic benefits for the offspring, based on these morphological traits, at least at the standard rearing temperature. Whether females actually exhibit such mating preferences for the traits measured here, requires experimental testing. For example, despite the existence of the non-additive genetic variance required for compatibility models to operate, questions must remain about the significance of mate choice based on compatibility in driving trait evolution, at least in the context of sexually dimorphic morphologies, such as body and pronotum size. This is because mate choice for compatibility will exert balancing selection on these traits, whereas sexual dimorphism would seem most likely to evolve under sex-specific directional selection. Finally, we note that the relevance of our findings to natural populations of crickets will also hinge on the effectiveness by which crickets, when developing in their natural habitat, can mitigate the effects of environmental fluctuations of the magnitude encountered in our study via microhabitat selection.

## Authors' contributions

MN, DKD and LWS designed the experiment, MN and DKD conducted the lab work. MN measured morphologies, MN conducted statistical analyses. MN, DKD, and LWS wrote the manuscript. All authors read and approved the final manuscript. 
